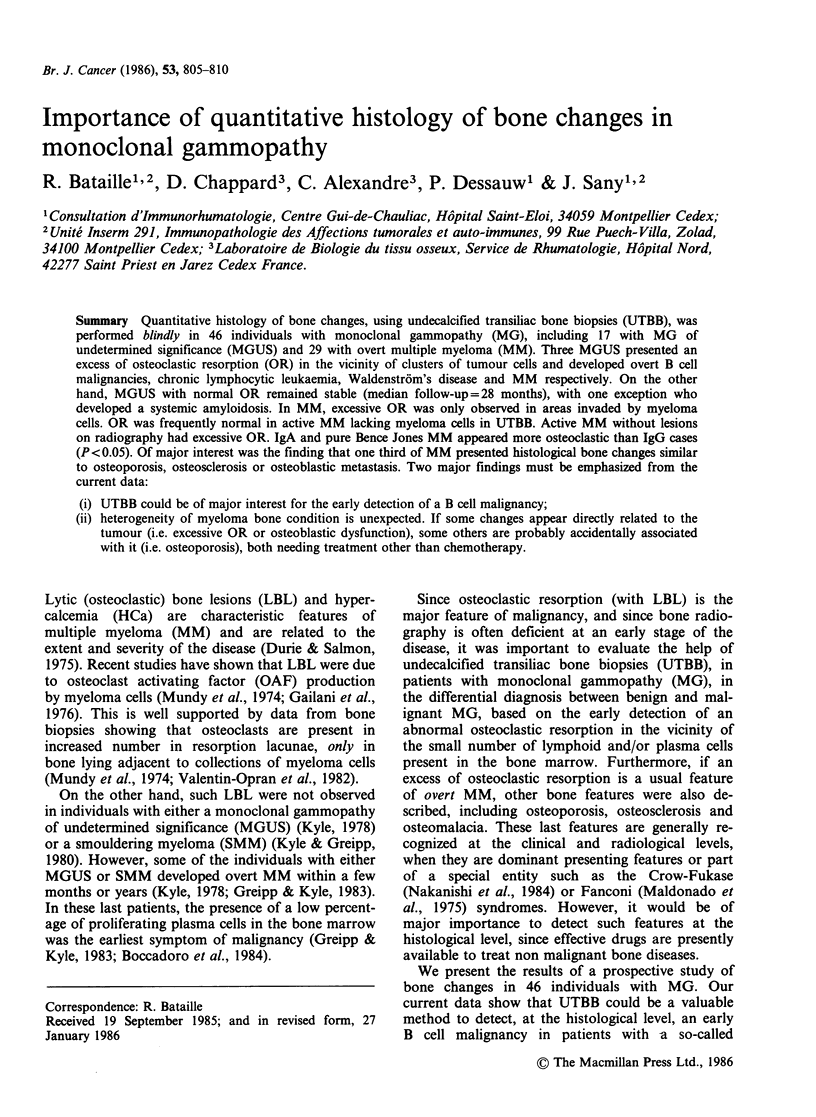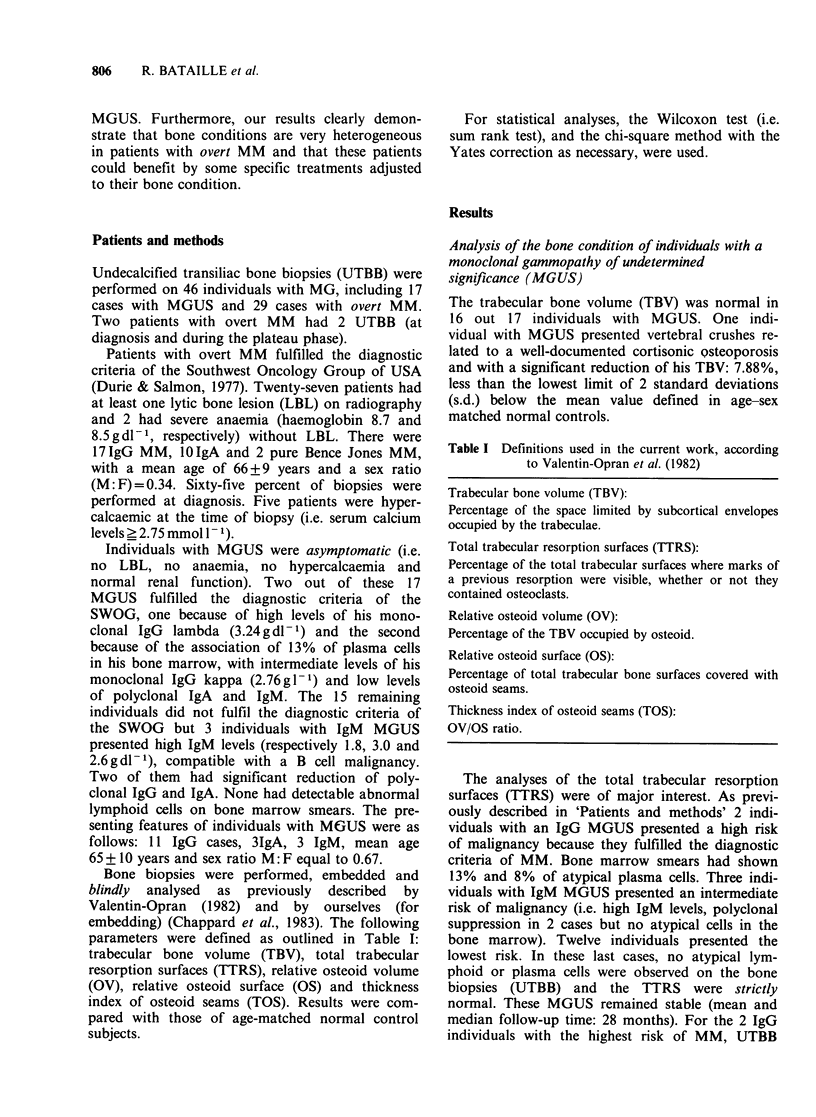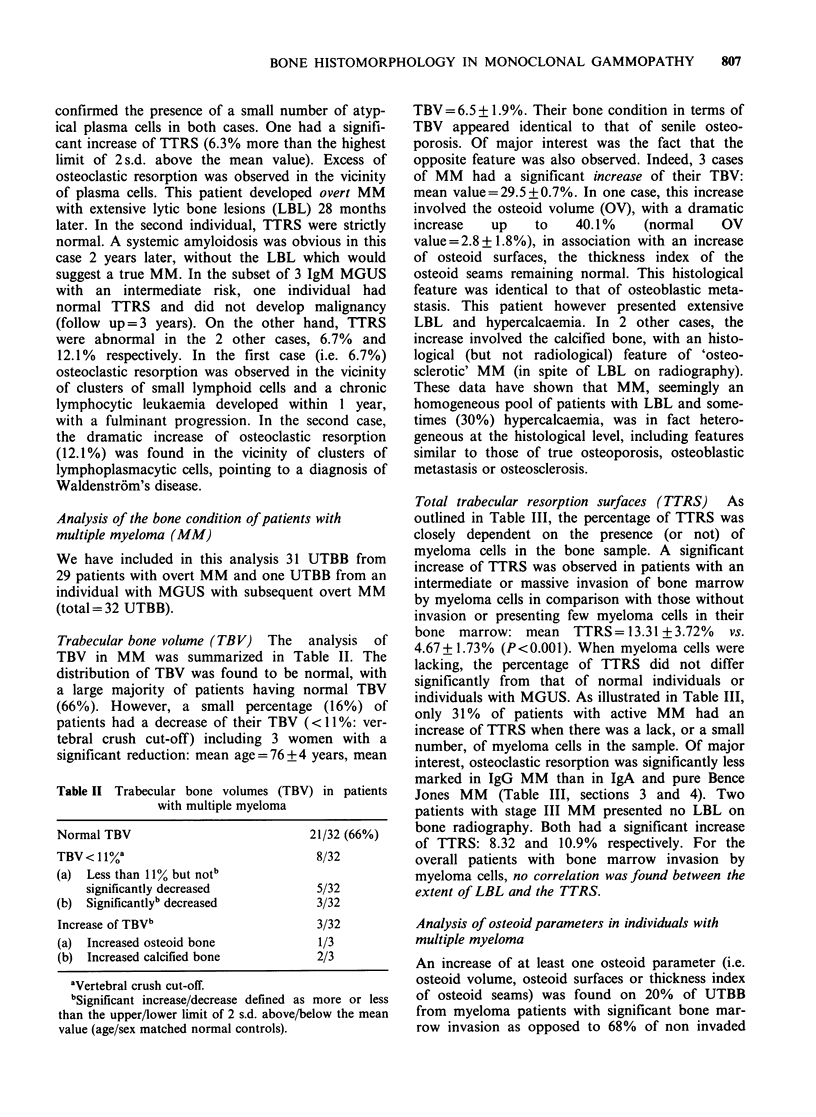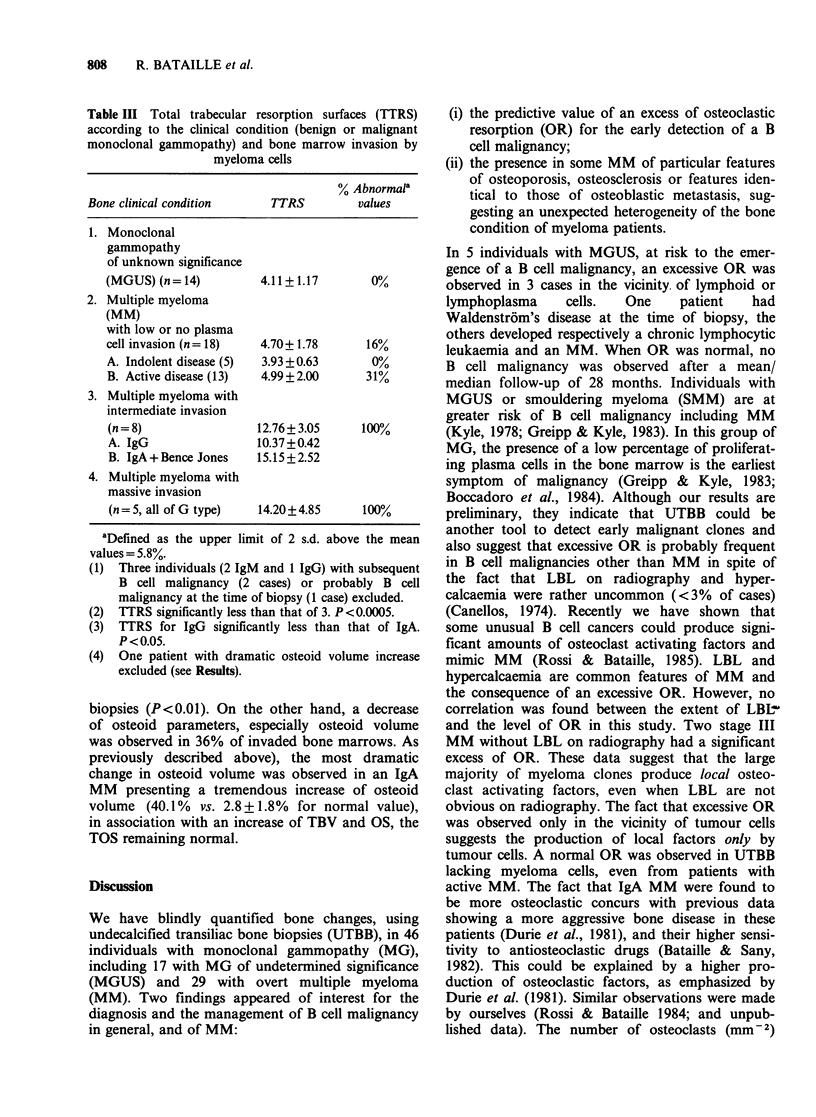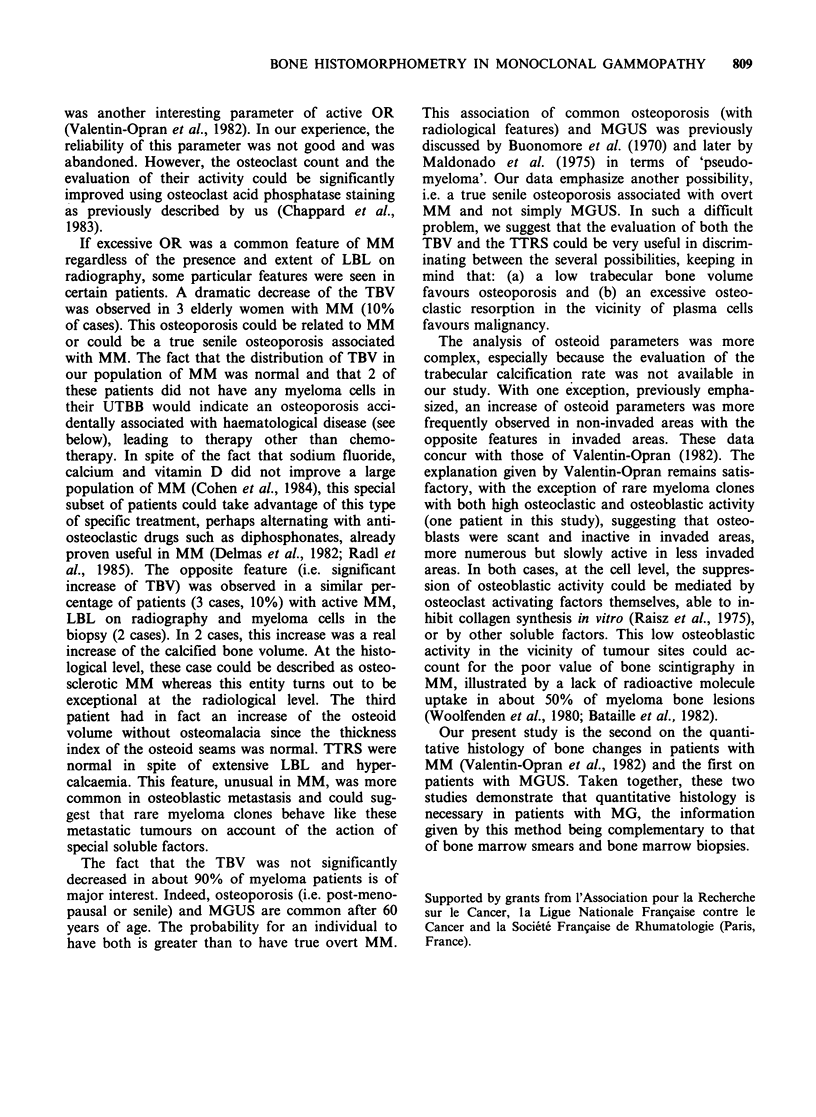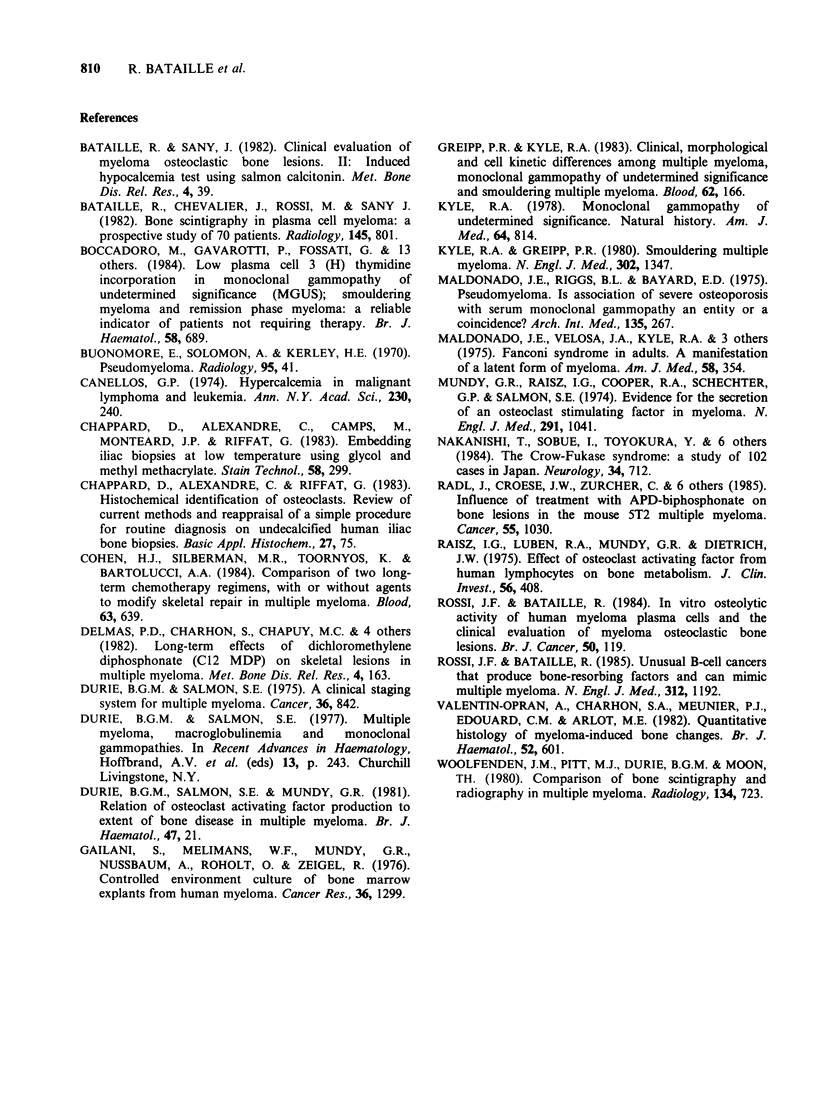# Importance of quantitative histology of bone changes in monoclonal gammopathy.

**DOI:** 10.1038/bjc.1986.136

**Published:** 1986-06

**Authors:** R. Bataille, D. Chappard, C. Alexandre, P. Dessauw, J. Sany

## Abstract

Quantitative histology of bone changes, using undecalcified transiliac bone biopsies (UTBB), was performed blindly in 46 individuals with monoclonal gammopathy (MG), including 17 with MG of undetermined significance (MGUS) and 29 with overt multiple myeloma (MM). Three MGUS presented an excess of osteoclastic resorption (OR) in the vicinity of clusters of tumour cells and developed overt B cell malignancies, chronic lymphocytic leukaemia, Waldenström's disease and MM respectively. On the other hand, MGUS with normal OR remained stable (median follow-up = 28 months), with one exception who developed a systemic amyloidosis. In MM, excessive OR was only observed in areas invaded by myeloma cells. OR was frequently normal in active MM lacking myeloma cells in UTBB. Active MM without lesions on radiography had excessive OR. IgA and pure Bence Jones MM appeared more osteoclastic than IgG cases (P less than 0.05). Of major interest was the finding that one third of MM presented histological bone changes similar to osteoporosis, osteosclerosis or osteoblastic metastasis. Two major findings must be emphasized from the current data: UTBB could be of major interest for the early detection of a B cell malignancy; heterogeneity of myeloma bone condition is unexpected. If some changes appear directly related to the tumour (i.e. excessive OR or osteoblastic dysfunction), some others are probably accidentally associated with it (i.e. osteoporosis), both needing treatment other than chemotherapy.


*Br. J. Cancer* (1986), **53**, 805–810

### Importance of quantitative histology of bone changes in monoclonal gammopathy

R. Bataille[1,2], D. Chappard[3], C. Alexandre[3], P. Dessauw[1] & J. Sany[1,2]

[1]*Consultation d'Immunorhumatologie, Centre Gui-de-Chauliac, Hôpital Saint-Eloi, 34059 Montpellier Cedex;* [2]*Unité Inserm 291, Immunopathologie des Affections tumorales et auto-immunes, 99 Rue Puech-Villa, Zolad, 34100 Montpellier Cedex;* [3]*Laboratoire de Biologie du tissu osseux, Service de Rhumatologie, Hôpital Nord, 42277 Saint Priest en Jarez Cedex France.*

**Summary** Quantitative histology of bone changes, using undecalcified transiliac bone biopsies (UTBB), was performed *blindly* in 46 individuals with monoclonal gammopathy (MG), including 17 with MG of undetermined significance (MGUS) and 29 with overt multiple myeloma (MM). Three MGUS presented an excess of osteoclastic resorption (OR) in the vicinity of clusters of tumour cells and developed overt B cell malignancies, chronic lymphocytic leukaemia, Waldenström's disease and MM respectively. On the other hand, MGUS with normal OR remained stable (median follow-up = 28 months), with one exception who developed a systemic amyloidosis. In MM, excessive OR was only observed in areas invaded by myeloma cells. OR was frequently normal in active MM lacking myeloma cells in UTBB. Active MM without lesions on radiography had excessive OR. IgA and pure Bence Jones MM appeared more osteoclastic than IgG cases ($P < 0.05$). Of major interest was the finding that one third of MM presented histological bone changes similar to osteoporosis, osteosclerosis or osteoblastic metastasis. Two major findings must be emphasized from the current data:

(i) UTBB could be of major interest for the early detection of a B cell malignancy;
(ii) heterogeneity of myeloma bone condition is unexpected. If some changes appear directly related to the tumour (i.e. excessive OR or osteoblastic dysfunction), some others are probably accidentally associated with it (i.e. osteoporosis), both needing treatment other than chemotherapy.

Lytic (osteoclastic) bone lesions (LBL) and hypercalcemia (HCa) are characteristic features of multiple myeloma (MM) and are related to the extent and severity of the disease (Durie & Salmon, 1975). Recent studies have shown that LBL were due to osteoclast activating factor (OAF) production by myeloma cells (Mundy *et al.*, 1974; Gailani *et al.*, 1976). This is well supported by data from bone biopsies showing that osteoclasts are present in increased number in resorption lacunae, *only* in bone lying adjacent to collections of myeloma cells (Mundy *et al.*, 1974; Valentin-Opran *et al.*, 1982).

On the other hand, such LBL were not observed in individuals with either a monoclonal gammopathy of undetermined significance (MGUS) (Kyle, 1978) or a smouldering myeloma (SMM) (Kyle & Greipp, 1980). However, some of the individuals with either MGUS or SMM developed overt MM within a few months or years (Kyle, 1978; Greipp & Kyle, 1983). In these last patients, the presence of a low percentage of proliferating plasma cells in the bone marrow was the earliest symptom of malignancy (Greipp & Kyle, 1983; Boccadoro *et al.*, 1984).

Correspondence: R. Bataille
Received 19 September 1985; and in revised form, 27 January 1986

Since osteoclastic resorption (with LBL) is the major feature of malignancy, and since bone radiography is often deficient at an early stage of the disease, it was important to evaluate the help of undecalcified transiliac bone biopsies (UTBB), in patients with monoclonal gammopathy (MG), in the differential diagnosis between benign and malignant MG, based on the early detection of an abnormal osteoclastic resorption in the vicinity of the small number of lymphoid and/or plasma cells present in the bone marrow. Furthermore, if an excess of osteoclastic resorption is a usual feature of *overt* MM, other bone features were also described, including osteoporosis, osteosclerosis and osteomalacia. These last features are generally recognized at the clinical and radiological levels, when they are dominant presenting features or part of a special entity such as the Crow-Fukase (Nakanishi *et al.*, 1984) or Fanconi (Maldonado *et al.*, 1975) syndromes. However, it would be of major importance to detect such features at the histological level, since effective drugs are presently available to treat non malignant bone diseases.

We present the results of a prospective study of bone changes in 46 individuals with MG. Our current data show that UTBB could be a valuable method to detect, at the histological level, an early B cell malignancy in patients with a so-called

© The Macmillan Press Ltd., 1986

MGUS. Furthermore, our results clearly demonstrate that bone conditions are very heterogeneous in patients with *overt* MM and that these patients could benefit by some specific treatments adjusted to their bone condition.

### Patients and methods

Undecalcified transiliac bone biopsies (UTBB) were performed on 46 individuals with MG, including 17 cases with MGUS and 29 cases with *overt* MM. Two patients with overt MM had 2 UTBB (at diagnosis and during the plateau phase).

Patients with overt MM fulfilled the diagnostic criteria of the Southwest Oncology Group of USA (Durie & Salmon, 1977). Twenty-seven patients had at least one lytic bone lesion (LBL) on radiography and 2 had severe anaemia (haemoglobin 8.7 and $8.5\,g\,dl^{-1}$, respectively) without LBL. There were 17 IgG MM, 10 IgA and 2 pure Bence Jones MM, with a mean age of $66 \pm 9$ years and a sex ratio (M:F) = 0.34. Sixty-five percent of biopsies were performed at diagnosis. Five patients were hypercalcaemic at the time of biopsy (i.e. serum calcium levels $\geq 2.75\,mmol\,l^{-1}$).

Individuals with MGUS were *asymptomatic* (i.e. no LBL, no anaemia, no hypercalcaemia and normal renal function). Two out of these 17 MGUS fulfilled the diagnostic criteria of the SWOG, one because of high levels of his monoclonal IgG lambda ($3.24\,g\,dl^{-1}$) and the second because of the association of 13% of plasma cells in his bone marrow, with intermediate levels of his monoclonal IgG kappa ($2.76\,g\,l^{-1}$) and low levels of polyclonal IgA and IgM. The 15 remaining individuals did not fulfil the diagnostic criteria of the SWOG but 3 individuals with IgM MGUS presented high IgM levels (respectively 1.8, 3.0 and $2.6\,g\,dl^{-1}$), compatible with a B cell malignancy. Two of them had significant reduction of polyclonal IgG and IgA. None had detectable abnormal lymphoid cells on bone marrow smears. The presenting features of individuals with MGUS were as follows: 11 IgG cases, 3IgA, 3 IgM, mean age $65 \pm 10$ years and sex ratio M:F equal to 0.67.

Bone biopsies were performed, embedded and *blindly* analysed as previously described by Valentin-Opran (1982) and by ourselves (for embedding) (Chappard *et al.*, 1983). The following parameters were defined as outlined in Table I: trabecular bone volume (TBV), total trabecular resorption surfaces (TTRS), relative osteoid volume (OV), relative osteoid surface (OS) and thickness index of osteoid seams (TOS). Results were compared with those of age-matched normal control subjects.

For statistical analyses, the Wilcoxon test (i.e. sum rank test), and the chi-square method with the Yates correction as necessary, were used.

### Results

### *Analysis of the bone condition of individuals with a monoclonal gammopathy of undetermined significance (MGUS)*

The trabecular bone volume (TBV) was normal in 16 out 17 individuals with MGUS. One individual with MGUS presented vertebral crushes related to a well-documented cortisonic osteoporosis and with a significant reduction of his TBV: 7.88%, less than the lowest limit of 2 standard deviations (s.d.) below the mean value defined in age–sex matched normal controls.

**Table I** Definitions used in the current work, according to Valentin-Opran *et al.* (1982)

| |
|---|
| Trabecular bone volume (TBV): |
| Percentage of the space limited by subcortical envelopes occupied by the trabeculae. |
| Total trabecular resorption surfaces (TTRS): |
| Percentage of the total trabecular surfaces where marks of a previous resorption were visible, whether or not they contained osteoclasts. |
| Relative osteoid volume (OV): |
| Percentage of the TBV occupied by osteoid. |
| Relative osteoid surface (OS): |
| Percentage of total trabecular bone surfaces covered with osteoid seams. |
| Thickness index of osteoid seams (TOS): |
| OV/OS ratio. |

The analyses of the total trabecular resorption surfaces (TTRS) were of major interest. As previously described in 'Patients and methods' 2 individuals with an IgG MGUS presented a high risk of malignancy because they fulfilled the diagnostic criteria of MM. Bone marrow smears had shown 13% and 8% of atypical plasma cells. Three individuals with IgM MGUS presented an intermediate risk of malignancy (i.e. high IgM levels, polyclonal suppression in 2 cases but no atypical cells in the bone marrow). Twelve individuals presented the lowest risk. In these last cases, no atypical lymphoid or plasma cells were observed on the bone biopsies (UTBB) and the TTRS were *strictly* normal. These MGUS remained stable (mean and median follow-up time: 28 months). For the 2 IgG individuals with the highest risk of MM, UTBB

confirmed the presence of a small number of atypical plasma cells in both cases. One had a significant increase of TTRS (6.3% more than the highest limit of 2 s.d. above the mean value). Excess of osteoclastic resorption was observed in the vicinity of plasma cells. This patient developed *overt* MM with extensive lytic bone lesions (LBL) 28 months later. In the second individual, TTRS were strictly normal. A systemic amyloidosis was obvious in this case 2 years later, without the LBL which would suggest a true MM. In the subset of 3 IgM MGUS with an intermediate risk, one individual had normal TTRS and did not develop malignancy (follow up = 3 years). On the other hand, TTRS were abnormal in the 2 other cases, 6.7% and 12.1% respectively. In the first case (i.e. 6.7%) osteoclastic resorption was observed in the vicinity of clusters of small lymphoid cells and a chronic lymphocytic leukaemia developed within 1 year, with a fulminant progression. In the second case, the dramatic increase of osteoclastic resorption (12.1%) was found in the vicinity of clusters of lymphoplasmacytic cells, pointing to a diagnosis of Waldenström's disease.

### *Analysis of the bone condition of patients with multiple myeloma (MM)*

We have included in this analysis 31 UTBB from 29 patients with overt MM and one UTBB from an individual with MGUS with subsequent overt MM (total = 32 UTBB).

*Trabecular bone volume (TBV)* The analysis of TBV in MM was summarized in Table II. The distribution of TBV was found to be normal, with a large majority of patients having normal TBV (66%). However, a small percentage (16%) of patients had a decrease of their TBV (<11%: vertebral crush cut-off) including 3 women with a significant reduction: mean age = $76 \pm 4$ years, mean TBV = $6.5 \pm 1.9$%. Their bone condition in terms of TBV appeared identical to that of senile osteoporosis. Of major interest was the fact that the opposite feature was also observed. Indeed, 3 cases of MM had a significant *increase* of their TBV: mean value = $29.5 \pm 0.7$%. In one case, this increase involved the osteoid volume (OV), with a dramatic increase up to 40.1% (normal OV value = $2.8 \pm 1.8$%), in association with an increase of osteoid surfaces, the thickness index of the osteoid seams remaining normal. This histological feature was identical to that of osteoblastic metastasis. This patient however presented extensive LBL and hypercalcaemia. In 2 other cases, the increase involved the calcified bone, with an histological (but not radiological) feature of 'osteosclerotic' MM (in spite of LBL on radiography). These data have shown that MM, seemingly an homogeneous pool of patients with LBL and sometimes (30%) hypercalcaemia, was in fact heterogeneous at the histological level, including features similar to those of true osteoporosis, osteoblastic metastasis or osteosclerosis.

**Table II** Trabecular bone volumes (TBV) in patients with multiple myeloma

| | |
|---|---|
| Normal TBV | 21/32 (66%) |
| TBV < 11%[a] | 8/32 |
| (a) Less than 11% but not[b] significantly decreased | 5/32 |
| (b) Significantly[b] decreased | 3/32 |
| Increase of TBV[b] | 3/32 |
| (a) Increased osteoid bone | 1/3 |
| (b) Increased calcified bone | 2/3 |

[a]Vertebral crush cut-off.
[b]Significant increase/decrease defined as more or less than the upper/lower limit of 2 s.d. above/below the mean value (age/sex matched normal controls).

*Total trabecular resorption surfaces (TTRS)* As outlined in Table III, the percentage of TTRS was closely dependent on the presence (or not) of myeloma cells in the bone sample. A significant increase of TTRS was observed in patients with an intermediate or massive invasion of bone marrow by myeloma cells in comparison with those without invasion or presenting few myeloma cells in their bone marrow: mean TTRS = $13.31 \pm 3.72$% *vs.* $4.67 \pm 1.73$% ($P < 0.001$). When myeloma cells were lacking, the percentage of TTRS did not differ significantly from that of normal individuals or individuals with MGUS. As illustrated in Table III, only 31% of patients with active MM had an increase of TTRS when there was a lack, or a small number, of myeloma cells in the sample. Of major interest, osteoclastic resorption was significantly less marked in IgG MM than in IgA and pure Bence Jones MM (Table III, sections 3 and 4). Two patients with stage III MM presented no LBL on bone radiography. Both had a significant increase of TTRS: 8.32 and 10.9% respectively. For the overall patients with bone marrow invasion by myeloma cells, *no correlation was found between the extent of LBL and the TTRS.*

### *Analysis of osteoid parameters in individuals with multiple myeloma*

An increase of at least one osteoid parameter (i.e. osteoid volume, osteoid surfaces or thickness index of osteoid seams) was found on 20% of UTBB from myeloma patients with significant bone marrow invasion as opposed to 68% of non invaded

**Table III** Total trabecular resorption surfaces (TTRS) according to the clinical condition (benign or malignant monoclonal gammopathy) and bone marrow invasion by myeloma cells

| *Bone clinical condition* | *TTRS* | *% Abnormal*[a] *values* |
|---|---|---|
| 1. Monoclonal gammopathy of unknown significance (MGUS) ($n=14$) | 4.11±1.17 | 0% |
| 2. Multiple myeloma (MM) with low or no plasma cell invasion ($n=18$) | 4.70±1.78 | 16% |
| A. Indolent disease (5) | 3.93±0.63 | 0% |
| B. Active disease (13) | 4.99±2.00 | 31% |
| 3. Multiple myeloma with intermediate invasion ($n=8$) | 12.76±3.05 | 100% |
| A. IgG | 10.37±0.42 | |
| B. IgA+Bence Jones | 15.15±2.52 | |
| 4. Multiple myeloma with massive invasion ($n=5$, all of G type) | 14.20±4.85 | 100% |

[a]Defined as the upper limit of 2 s.d. above the mean values = 5.8%.

(1) Three individuals (2 IgM and 1 IgG) with subsequent B cell malignancy (2 cases) or probably B cell malignancy at the time of biopsy (1 case) excluded.

(2) TTRS significantly less than that of 3. $P<0.0005$.

(3) TTRS for IgG significantly less than that of IgA. $P<0.05$.

(4) One patient with dramatic osteoid volume increase excluded (see **Results**).

biopsies ($P<0.01$). On the other hand, a decrease of osteoid parameters, especially osteoid volume was observed in 36% of invaded bone marrows. As previously described above), the most dramatic change in osteoid volume was observed in an IgA MM presenting a tremendous increase of osteoid volume (40.1% *vs.* 2.8±1.8% for normal value), in association with an increase of TBV and OS, the TOS remaining normal.

### Discussion

We have blindly quantified bone changes, using undecalcified transiliac bone biopsies (UTBB), in 46 individuals with monoclonal gammopathy (MG), including 17 with MG of undetermined significance (MGUS) and 29 with overt multiple myeloma (MM). Two findings appeared of interest for the diagnosis and the management of B cell malignancy in general, and of MM:

(i) the predictive value of an excess of osteoclastic resorption (OR) for the early detection of a B cell malignancy;
(ii) the presence in some MM of particular features of osteoporosis, osteosclerosis or features identical to those of osteoblastic metastasis, suggesting an unexpected heterogeneity of the bone condition of myeloma patients.

In 5 individuals with MGUS, at risk to the emergence of a B cell malignancy, an excessive OR was observed in 3 cases in the vicinity of lymphoid or lymphoplasma cells. One patient had Waldenström's disease at the time of biopsy, the others developed respectively a chronic lymphocytic leukaemia and an MM. When OR was normal, no B cell malignancy was observed after a mean/median follow-up of 28 months. Individuals with MGUS or smouldering myeloma (SMM) are at greater risk of B cell malignancy including MM (Kyle, 1978; Greipp & Kyle, 1983). In this group of MG, the presence of a low percentage of proliferating plasma cells in the bone marrow is the earliest symptom of malignancy (Greipp & Kyle, 1983; Boccadoro *et al.*, 1984). Although our results are preliminary, they indicate that UTBB could be another tool to detect early malignant clones and also suggest that excessive OR is probably frequent in B cell malignancies other than MM in spite of the fact that LBL on radiography and hypercalcaemia were rather uncommon (<3% of cases) (Canellos, 1974). Recently we have shown that some unusual B cell cancers could produce significant amounts of osteoclast activating factors and mimic MM (Rossi & Bataille, 1985). LBL and hypercalcaemia are common features of MM and the consequence of an excessive OR. However, no correlation was found between the extent of LBL and the level of OR in this study. Two stage III MM without LBL on radiography had a significant excess of OR. These data suggest that the large majority of myeloma clones produce *local* osteoclast activating factors, even when LBL are not obvious on radiography. The fact that excessive OR was observed only in the vicinity of tumour cells suggests the production of local factors *only* by tumour cells. A normal OR was observed in UTBB lacking myeloma cells, even from patients with active MM. The fact that IgA MM were found to be more osteoclastic concurs with previous data showing a more aggressive bone disease in these patients (Durie *et al.*, 1981), and their higher sensitivity to antiosteoclastic drugs (Bataille & Sany, 1982). This could be explained by a higher production of osteoclastic factors, as emphasized by Durie *et al.* (1981). Similar observations were made by ourselves (Rossi & Bataille 1984; and unpublished data). The number of osteoclasts ($mm^{-2}$)

was another interesting parameter of active OR (Valentin-Opran *et al.*, 1982). In our experience, the reliability of this parameter was not good and was abandoned. However, the osteoclast count and the evaluation of their activity could be significantly improved using osteoclast acid phosphatase staining as previously described by us (Chappard *et al.*, 1983).

If excessive OR was a common feature of MM regardless of the presence and extent of LBL on radiography, some particular features were seen in certain patients. A dramatic decrease of the TBV was observed in 3 elderly women with MM (10% of cases). This osteoporosis could be related to MM or could be a true senile osteoporosis associated with MM. The fact that the distribution of TBV in our population of MM was normal and that 2 of these patients did not have any myeloma cells in their UTBB would indicate an osteoporosis accidentally associated with haematological disease (see below), leading to therapy other than chemotherapy. In spite of the fact that sodium fluoride, calcium and vitamin D did not improve a large population of MM (Cohen *et al.*, 1984), this special subset of patients could take advantage of this type of specific treatment, perhaps alternating with anti-osteoclastic drugs such as diphosphonates, already proven useful in MM (Delmas *et al.*, 1982; Radl *et al.*, 1985). The opposite feature (i.e. significant increase of TBV) was observed in a similar percentage of patients (3 cases, 10%) with active MM, LBL on radiography and myeloma cells in the biopsy (2 cases). In 2 cases, this increase was a real increase of the calcified bone volume. At the histological level, these case could be described as osteosclerotic MM whereas this entity turns out to be exceptional at the radiological level. The third patient had in fact an increase of the osteoid volume without osteomalacia since the thickness index of the osteoid seams was normal. TTRS were normal in spite of extensive LBL and hypercalcaemia. This feature, unusual in MM, was more common in osteoblastic metastasis and could suggest that rare myeloma clones behave like these metastatic tumours on account of the action of special soluble factors.

The fact that the TBV was not significantly decreased in about 90% of myeloma patients is of major interest. Indeed, osteoporosis (i.e. post-menopausal or senile) and MGUS are common after 60 years of age. The probability for an individual to have both is greater than to have true overt MM. This association of common osteoporosis (with radiological features) and MGUS was previously discussed by Buonomore *et al.* (1970) and later by Maldonado *et al.* (1975) in terms of 'pseudo-myeloma'. Our data emphasize another possibility, i.e. a true senile osteoporosis associated with overt MM and not simply MGUS. In such a difficult problem, we suggest that the evaluation of both the TBV and the TTRS could be very useful in discriminating between the several possibilities, keeping in mind that: (a) a low trabecular bone volume favours osteoporosis and (b) an excessive osteoclastic resorption in the vicinity of plasma cells favours malignancy.

The analysis of osteoid parameters was more complex, especially because the evaluation of the trabecular calcification rate was not available in our study. With one exception, previously emphasized, an increase of osteoid parameters was more frequently observed in non-invaded areas with the opposite features in invaded areas. These data concur with those of Valentin-Opran (1982). The explanation given by Valentin-Opran remains satisfactory, with the exception of rare myeloma clones with both high osteoclastic and osteoblastic activity (one patient in this study), suggesting that osteoblasts were scant and inactive in invaded areas, more numerous but slowly active in less invaded areas. In both cases, at the cell level, the suppression of osteoblastic activity could be mediated by osteoclast activating factors themselves, able to inhibit collagen synthesis *in vitro* (Raisz *et al.*, 1975), or by other soluble factors. This low osteoblastic activity in the vicinity of tumour sites could account for the poor value of bone scintigraphy in MM, illustrated by a lack of radioactive molecule uptake in about 50% of myeloma bone lesions (Woolfenden *et al.*, 1980; Bataille *et al.*, 1982).

Our present study is the second on the quantitative histology of bone changes in patients with MM (Valentin-Opran *et al.*, 1982) and the first on patients with MGUS. Taken together, these two studies demonstrate that quantitative histology is necessary in patients with MG, the information given by this method being complementary to that of bone marrow smears and bone marrow biopsies.

Supported by grants from l'Association pour la Recherche sur le Cancer, la Ligue Nationale Française contre le Cancer and la Société Française de Rhumatologie (Paris, France).

### References

BATAILLE, R. & SANY, J. (1982). Clinical evaluation of myeloma osteoclastic bone lesions. II: Induced hypocalcemia test using salmon calcitonin. *Met. Bone Dis. Rel. Res.*, **4**, 39.

BATAILLE, R., CHEVALIER, J., ROSSI, M. & SANY J. (1982). Bone scintigraphy in plasma cell myeloma: a prospective study of 70 patients. *Radiology*, **145**, 801.

BOCCADORO, M., GAVAROTTI, P., FOSSATI, G. & 13 others. (1984). Low plasma cell 3 (H) thymidine incorporation in monoclonal gammopathy of undetermined significance (MGUS); smouldering myeloma and remission phase myeloma: a reliable indicator of patients not requiring therapy. *Br. J. Haematol.*, **58**, 689.

BUONOMORE, E., SOLOMON, A. & KERLEY, H.E. (1970). Pseudomyeloma. *Radiology*, **95**, 41.

CANELLOS, G.P. (1974). Hypercalcemia in malignant lymphoma and leukemia. *Ann. N.Y. Acad. Sci.*, **230**, 240.

CHAPPARD, D., ALEXANDRE, C., CAMPS, M., MONTEARD, J.P. & RIFFAT, G. (1983). Embedding iliac biopsies at low temperature using glycol and methyl methacrylate. *Stain Technol.*, **58**, 299.

CHAPPARD, D., ALEXANDRE, C. & RIFFAT, G. (1983). Histochemical identification of osteoclasts. Review of current methods and reappraisal of a simple procedure for routine diagnosis on undecalcified human iliac bone biopsies. *Basic Appl. Histochem.*, **27**, 75.

COHEN, H.J., SILBERMAN, M.R., TOORNYOS, K. & BARTOLUCCI, A.A. (1984). Comparison of two long-term chemotherapy regimens, with or without agents to modify skeletal repair in multiple myeloma. *Blood*, **63**, 639.

DELMAS, P.D., CHARHON, S., CHAPUY, M.C. & 4 others (1982). Long-term effects of dichloromethylene diphosphonate (C12 MDP) on skeletal lesions in multiple myeloma. *Met. Bone Dis. Rel. Res.*, **4**, 163.

DURIE, B.G.M. & SALMON, S.E. (1975). A clinical staging system for multiple myeloma. *Cancer*, **36**, 842.

DURIE, B.G.M. & SALMON, S.E. (1977). Multiple myeloma, macroglobulinemia and monoclonal gammopathies. In *Recent Advances in Haematology*, Hoffbrand, A.V. *et al.* (eds) **13**, p. 243. Churchill Livingstone, N.Y.

DURIE, B.G.M., SALMON, S.E. & MUNDY, G.R. (1981). Relation of osteoclast activating factor production to extent of bone disease in multiple myeloma. *Br. J. Haematol.*, **47**, 21.

GAILANI, S., MELIMANS, W.F., MUNDY, G.R., NUSSBAUM, A., ROHOLT, O. & ZEIGEL, R. (1976). Controlled environment culture of bone marrow explants from human myeloma. *Cancer Res.*, **36**, 1299.

GREIPP, P.R. & KYLE, R.A. (1983). Clinical, morphological and cell kinetic differences among multiple myeloma, monoclonal gammopathy of undetermined significance and smouldering multiple myeloma. *Blood*, **62**, 166.

KYLE, R.A. (1978). Monoclonal gammopathy of undetermined significance. Natural history. *Am. J. Med.*, **64**, 814.

KYLE, R.A. & GREIPP, P.R. (1980). Smouldering multiple myeloma. *N. Engl. J. Med.*, **302**, 1347.

MALDONADO, J.E., RIGGS, B.L. & BAYARD, E.D. (1975). Pseudomyeloma. Is association of severe osteoporosis with serum monoclonal gammopathy an entity or a coincidence? *Arch. Int. Med.*, **135**, 267.

MALDONADO, J.E., VELOSA, J.A., KYLE, R.A. & 3 others (1975). Fanconi syndrome in adults. A manifestation of a latent form of myeloma. *Am. J. Med.*, **58**, 354.

MUNDY, G.R., RAISZ, I.G., COOPER, R.A., SCHECHTER, G.P. & SALMON, S.E. (1974). Evidence for the secretion of an osteoclast stimulating factor in myeloma. *N. Engl. J. Med.*, **291**, 1041.

NAKANISHI, T., SOBUE, I., TOYOKURA, Y. & 6 others (1984). The Crow-Fukase syndrome: a study of 102 cases in Japan. *Neurology*, **34**, 712.

RADL, J., CROESE, J.W., ZURCHER, C. & 6 others (1985). Influence of treatment with APD-biphosphonate on bone lesions in the mouse 5T2 multiple myeloma. *Cancer*, **55**, 1030.

RAISZ, I.G., LUBEN, R.A., MUNDY, G.R. & DIETRICH, J.W. (1975). Effect of osteoclast activating factor from human lymphocytes on bone metabolism. *J. Clin. Invest.*, **56**, 408.

ROSSI, J.F. & BATAILLE, R. (1984). In vitro osteolytic activity of human myeloma plasma cells and the clinical evaluation of myeloma osteoclastic bone lesions. *Br. J. Cancer*, **50**, 119.

ROSSI, J.F. & BATAILLE, R. (1985). Unusual B-cell cancers that produce bone-resorbing factors and can mimic multiple myeloma. *N. Engl. J. Med.*, **312**, 1192.

VALENTIN-OPRAN, A., CHARHON, S.A., MEUNIER, P.J., EDOUARD, C.M. & ARLOT, M.E. (1982). Quantitative histology of myeloma-induced bone changes. *Br. J. Haematol.*, **52**, 601.

WOOLFENDEN, J.M., PITT, M.J., DURIE, B.G.M. & MOON, TH. (1980). Comparison of bone scintigraphy and radiography in multiple myeloma. *Radiology*, **134**, 723.